# SOM Neural Network Fault Diagnosis Method of Polymerization Kettle Equipment Optimized by Improved PSO Algorithm

**DOI:** 10.1155/2014/937680

**Published:** 2014-07-24

**Authors:** Jie-sheng Wang, Shu-xia Li, Jie Gao

**Affiliations:** ^1^School of Electronic and Information Engineering, University of Science & Technology Liaoning, Anshan 114044, China; ^2^National Financial Security and System Equipment Engineering Research Center, University of Science & Technology Liaoning, Anshan 114044, China

## Abstract

For meeting the real-time fault diagnosis and the optimization monitoring requirements of the polymerization kettle in the polyvinyl chloride resin (PVC) production process, a fault diagnosis strategy based on the self-organizing map (SOM) neural network is proposed. Firstly, a mapping between the polymerization process data and the fault pattern is established by analyzing the production technology of polymerization kettle equipment. The particle swarm optimization (PSO) algorithm with a new dynamical adjustment method of inertial weights is adopted to optimize the structural parameters of SOM neural network. The fault pattern classification of the polymerization kettle equipment is to realize the nonlinear mapping from symptom set to fault set according to the given symptom set. Finally, the simulation experiments of fault diagnosis are conducted by combining with the industrial on-site historical data of the polymerization kettle and the simulation results show that the proposed PSO-SOM fault diagnosis strategy is effective.

## 1. Introduction

Polyvinyl chloride (PVC) is one of the five largest thermoplastic synthetic resins, and its production is second only to the polyethylene (PE) and polypropylene (PP). PVC is a kind of general colophony, which is good in quality and is widely used. It has good mechanical properties, ant chemical properties, and it is corrosion-resistant and difficult to burn [1]. With vinyl chloride monomer (VCM) as a raw material, the suspension method to produce polyvinyl chloride (PVC) resin is a kind of typical batch chemical production process. PVC polymerization process is a complex control system with multivariable, uncertain, nonlinear, and strong coupling. Polymerization kettle is the key equipment of the PVC production process, where vinyl chlorides go on the polymerization reaction to generate polyvinyl chloride [1]. Whether the polymerization kettle can run steadily is directly related to the working conditions of the PVC production device. On the other hand, the motor, reducer, and machine seal are key equipment to ensure that the polymerization kettle device runs normally. Once they failed to work, the serious losses will be brought to the PVC polymerizing process [2]. Therefore, the earlier diagnosis of the fault type and location of polymerization kettle can avoid the huge economic losses which are caused by the parking of polymerization kettle, which has the important practical significance to improve the product quality and reduce the production costs [3, 4].

Self-organizing map (SOM) neural network, also called as the Kohonen network, is a kind of the unsupervised learning network. Its main feature is the ability to automatically seek the essential attributes and the intrinsic rules of the training samples and to change neuron structure and network variables through the characteristics of self-adaptation and self-organizing [5–7]. In the network training process, the data only includes the input samples and there are no corresponding ideal output samples. Through the self-learning of network, the connection weights between neurons can be changed by the self-organization strategy to find the inherent relations among the input samples and complete the self-learning and automatic classification of the network.

SOM neural network is widely applied in the fault diagnosis field. The SOM neural network is established based on the adjustable kernel function method and the genetic algorithm (GA) is adopted to adjust the SOM neural network parameters to obtain better classification results than single kernel function [8]. A fault diagnosis method combining the wavelet packet analysis with SOM neural network is put forward. Firstly, the gear model is established by using the virtual prototype technology to simulate all kinds of faults. Then the wavelet packet analysis is used to extract energy characteristic. Finally, the SOM neural network is used to classify the fault data [9]. For the difficult identification problem of rock volcanic, an identification method of rock nature combining the principal component analysis method with SOM neural network is proposed [10]. In this paper, for meeting the real-time fault diagnosis and optimization monitoring requirements of polymerization kettle, a real-time fault diagnosis strategy of polymerization kettle based on SOM neural network is proposed. The improved PSO algorithm is adopted to optimize the structure parameters of SOM neural network. The simulation results verify the efficiency of the proposed fault diagnosis strategy.

The paper is organized as follows. In Section 2, the technique flowchart of the PVC polymerization process is introduced. The SOM neural network is presented in Section 3. In Section 4, the SOM neural network optimized by the improved PSO algorithm is introduced. The simulation experiments and results analysis are introduced in detail in Section 5. Finally, the conclusion illustrates the last part.

## 2. Polyvinyl Chloride (PVC) Polymerization Process

### 2.1. Technique Flowchart

**Figure 1:**
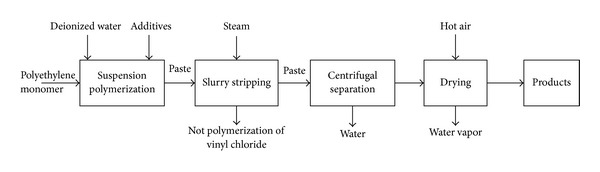
Flowchart of suspension polymerization.

Four methods (suspension polymerization, emulsion polymerization, bulk polymerization, and liquor polymerization) are usually used in the PVC polymerization process. Among them, the suspension polymerization is one of the most widely used methods, whose technique flowchart is shown in Figure 1 [3].

**Figure 2:**
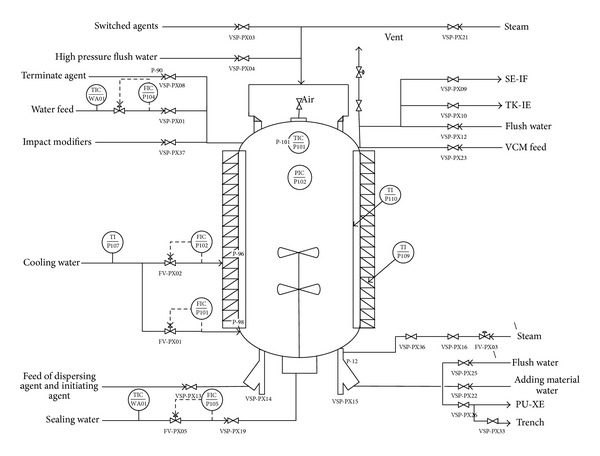
Technique flowchart of polymerization kettle.

Firstly, the suspending agent and deionizer water are fed into the polymerization kettle. Then the initiator is added and the polymerization kettle is sealed. The oxygen in the material and the air in the polymerization kettle are removed by vacuum. After adding the monomer vinyl chloride, the polymerization kettle starts to be stirred and heated. The temperature must be kept around 50°C and the pressure is maintained to 0.89~1.23 MPa. When the conversion ratio reaches about 70%, the pressure is reduced gradually. When the pressure drops to 0.13~0.48 MPa, the polymerization kettle reaction is terminated. After the transformation completed, the vinyl chloride monomer not being reacted are pulled out. The remaining slurry is carried out the stripping process to recycle the pulled vinyl chloride monomer. Then a centrifugal separation is used on the stripped slurry. When the water content reaches around 25%, the slurry is put into the dryer until the water content reaches about 0.3%~0.4%. The typical technique process of PVC polymerization kettle is shown in Figure 2 [3].

In PVC polymerization process, various raw materials and additives are added to the reaction kettle, which are evenly dispersed under the mixing action. Then the suitable amounts of the initiators are added to the kettle and start to react. The cooling water is constantly poured into the jacket and baffle of the reaction kettle to remove the reaction heat. The reaction will be terminated and the final products are obtained when the conversion ratio of the vinyl chloride (VCM) reaches a certain value and a proper pressure drop appears. Finally, after the reaction completed and VCM contained in slurry separated by the stripping technique, the remaining slurry is fed into the drying process for dewatering and drying.

### 2.2. Structure of Fault Diagnosis System and Information Table

**Figure 3:**
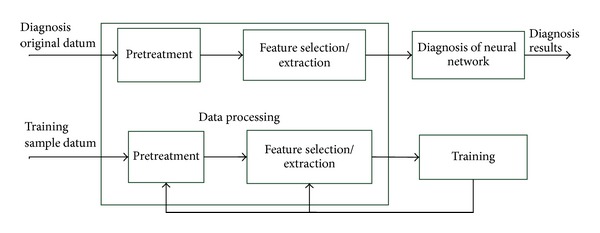
Structure of neural network fault diagnosis system.

The structure of the proposed polymerization kettle neural network fault diagnosis system is shown in Figure 3. Firstly, a set of fault samples are used to train the neural network to obtain the structure parameters. Then the pattern classification of faults is to realize the nonlinear mapping from symptom set to fault set according to a given set of symptoms.

The proposed fault diagnosis system applied a certain 70 M^3^ polymerization kettle from a large chemical company with the measured data. The main parameters of the polymerization kettle fault diagnosis are shown in Table 1.

It can be seen from Table 1 that the main parameters of polymeric kettle include stirring speed (r/min), stirring electric current (A), polymeric kettle pressure (MPa), polymeric kettle temperature (°C), mechanical seal pressure (MPa), and mechanical seal temperature (°C). These six parameters of polymerization kettle are, respectively, noted as *a*, *b*, *c*, *d*, *e*, and *f*. The motor fault, shaft seal fault, the damage of sealing components, and the running smoothly are, respectively, represented by 1, 2, 3, and 4. Large amounts of on-spot data are collected from the PVC polymerization kettle as input samples and testing samples of the neural network fault diagnosis system. The history working data of polymeric kettle are shown in Table 2.

## 3. Self-Organizing Map (SOM) Neural Network

### 3.1. Structure of SOM Neural Network

**Figure 4:**
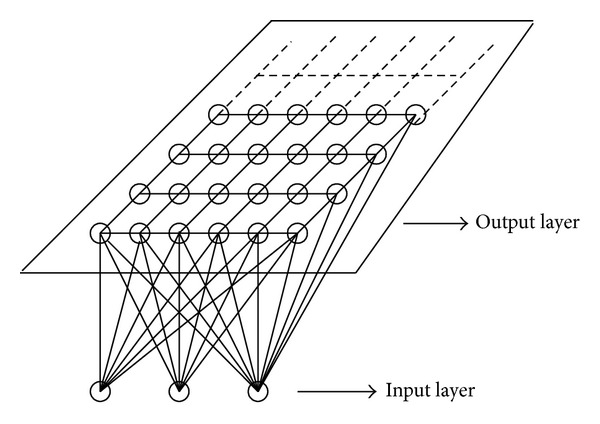
Structure of SOM neural network.

The structure of the self-organizing map (SOM) neural network is shown in Figure 4, which simulates the self-organizing feature map function of the human brain [11, 12].

SOM neural network is composed of the input layer and the output layer. The number of neurons in the input layer is determined by the number of feature vectors of input network. The input neurons as one dimensional matrix receive the input signals of network. Each neuron in the initialized output layer is connected with the adjacent neurons to form a two-dimension even distribution node matrix, which is called as transverse connection. The connection between the output layer and the input layer is realized with weights, which belongs to a two-way connection. When the training samples are fed into the input layer of SOM neural network, certain neurons in the output layer will get excited through the connection weights, but some neurons will be suppressed.

### 3.2. Principle of SOM Neural Network

#### 3.2.1. Similarity Measurement

SOM neural network adopts the distance between vectors to measure the similarity of input pattern vector. The two most commonly used methods are the Euclidean distance method and the cosine method.

(*1) Euclidean Distance Method*. Suppose *X*, *X*_*i*_ are two vectors, whose Euclidean distance is defined as follows:

(1) $$\begin{array}{c} d=||X-X_{i}||=\sqrt{\left(X-X_{i}\right){\left(X-X_{i}\right)}^{T}}. \end{array}$$

**Figure 5:**
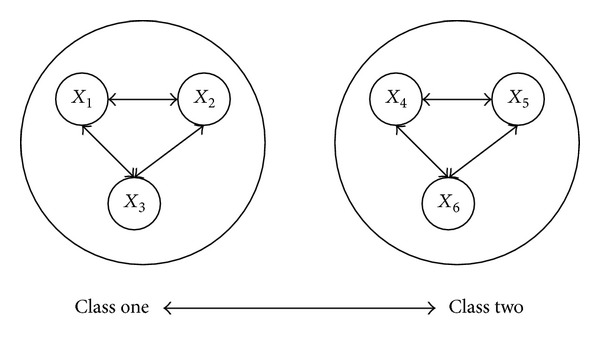
Data classification method based on Euclidean distance.

The smaller *d* is, the closer *X* is to *X*_*i*_, that is to say, the more similar they are. When *d* = 0, *X* = *X*_*i*_. With *d* = *T*(constant) as the criterion, a clustering analysis can be carried out with the input data. The data classification based on the Euclidean distance method is shown in Figure 5. Since *d*_12_, *d*_23_, *d*_31_ are all less than *T* and *d*_45_, *d*_56_, *d*_46_ are all less than *T*, while *d*_1*i*_ > *T*  (*i* = 4,5, 6), *d*_2*i*_ > *T*  (*i* = 4,5, 6), and *d*_3*i*_ > *T*  (*i* = 4,5, 6), hence the input data *X*_1_, *X*_2_, *X*_3_, *X*_4_, *X*_5_, *X*_6_ can be divided into two classes: Class 1 and Class 2.

(*2) Cosine Method*. Suppose *X*, *X*_*i*_ are two vectors; then the angle cosine between them is calculated as follows:

(2) $$\begin{array}{c} \cos\varphi =\frac{XX^{T}}{||X||||X_{i}||}. \end{array}$$

The smaller *d* is, the closer *X* is to *X*_*i*_, the more similar they are. When *φ* = 0, cos⁡*φ* = 1 and *X* = *X*_*i*_. Similarly, with *φ* = *φ*_0_, the input data can be carried out the clustering analysis.

#### 3.2.2. Competitive Learning Principle

The competitive learning rule is a lateral inhibition phenomenon of the nerve cells in the human body. That is to say, when a nerve cell generates excitement, it will produce inhibition effect on its surrounding nerve cells, among which the competition winning cell has the strongest inhibitory effect. This method is called “Winner-Take-All (WTA).” Its learning steps are described as follows.

(*1) Vector Normalization*. In practical problems, each component of the *n*-dimension variable *X* may have different unit. The purpose of vector normalization is to turn vectors into the unit vector with the invariant direction mold of 1. The vector normalization can be realized by the following equations:

(3) $$\begin{array}{c} \hat{X}=\frac{X}{||X||},\;\;\hat{W}=\frac{W_{j}}{||W_{j}||}. \end{array}$$

**Figure 6:**
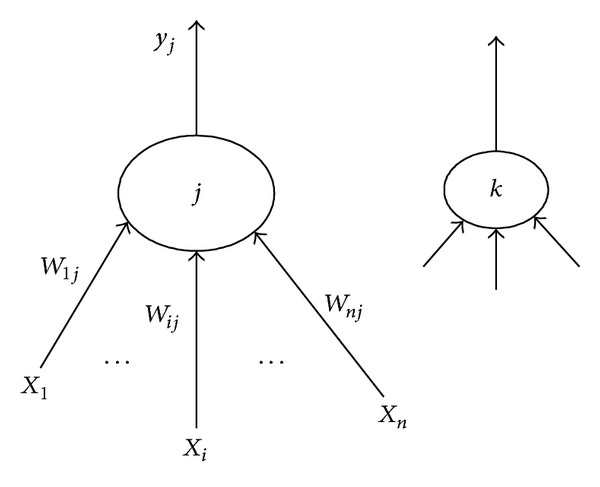
Vector normalization.

For the SOM neural network, the input vector *X* and the weight vector of each neuron in the output layer *w*_*j*_  (*j* = 1,2,…, *m*), shown in Figure 6, all are normalized to obtain $\hat{X}$ and $\hat{W_{j^{*}}}$.

(*2) Seek Winning Neurons*. The similarity between $\hat{X}$ and the weight vectors *w*_*j*_  (*j* = 1,2,…, *m*) corresponded by all neurons in the output layer is calculated and compared. The most similar neurons will get win, whose weight vector is denoted as $\hat{W_{j^{*}}}$:

(4) ||X⌢−  W⌢j∗||=min⁡j∈{1,2,…,n}{||X^−W^j||},||X^−W^j∗||=(X^−Wj∗)(X^−Wj∗)T=X^X^T−2W^j∗X^T+W^j∗W^j∗T=2(1−W^j∗X^T),Wj∗^XT^=max⁡j(Wj^XT^).

(*3) Network Output and Weight Adjustment*. According to the WTA learning law, the output of winning neuron is 1, and the rest of neurons are 0. That is to say,

(5) $$\begin{array}{c} y_{j}\left(t+1\right)=\{\begin{array}{cc} 1, & j=j^{*},\\ 0, & j\neq j^{*}. \end{array} \end{array}$$

Only the winning neuron has the right to adjust the weight vector *W*_j*_. The learning adjustment rule of weight vector is described as follows:

(6) $$\begin{array}{c} W_{j^{*}}\left(t+1\right)={\hat{W}}_{j^{*}}\left(t\right)+\Delta W_{j^{*}}={\hat{W}}_{j^{*}}\left(t\right)+\alpha \left(\hat{X}-{\hat{W}}_{j^{*}}\right),\\ W_{j}\left(t+1\right)={\hat{W}}_{j}\left(t\right),\;j\neq j^{*}, \end{array}$$

where 0 < *α* ≤ 1 is the learning rate. Generally, *α* decreases with the learning progress, so the degree of adjustment will be more and more tiny and finally tend to cluster centers.

(*4) Normalized Again.* The weight vectors that have been normalized are no longer unit vectors after being adjusted; hence, vectors that have been learnt and adjusted are given a normalized disposal again, until the learning rate *α* decays to zero.

### 3.3. Algorithm Procedure

**Figure 7:**
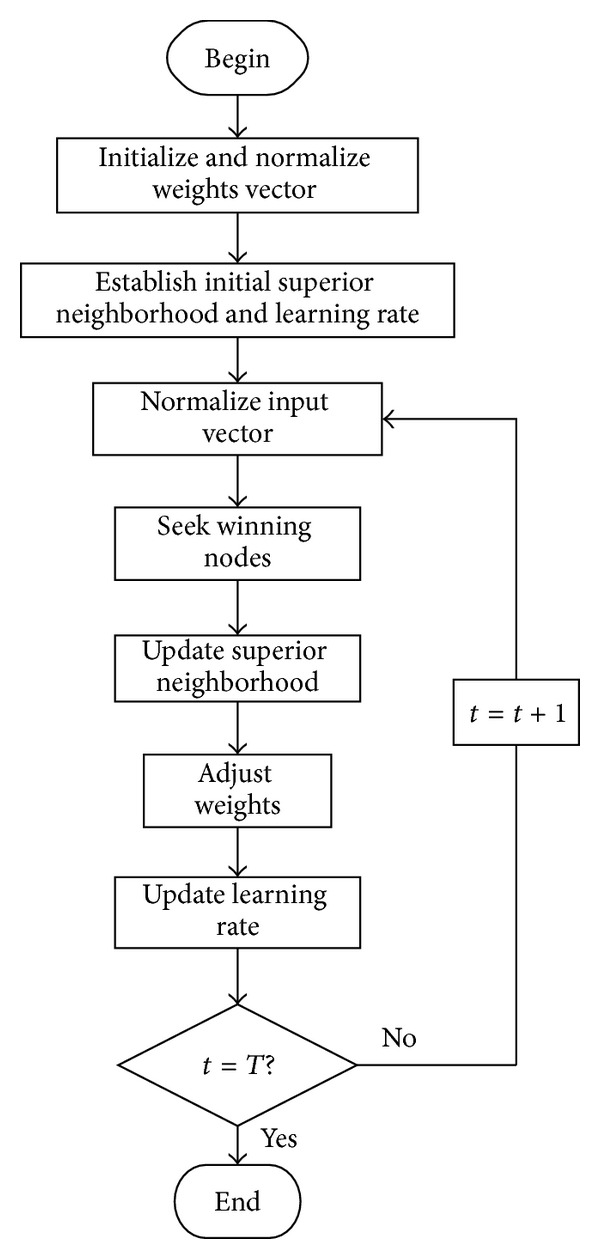
Flowchart of SOM algorithm.

The learning algorithm procedure of SOM neural network is shown in Figure 7.

The learning steps are described as follows.

**Step 1 (network initialization).**

Initialize the weights between the input layer and the mapping layer with random numbers. Initialize the connection weights between the input neurons and the output neurons. Select the “adjacent neuron” set *S*_*j*_ of the output neurons *j*, among which *S*_*j*_(0) represents the “adjacent neuron” set of neuron *j* at the time *t* = 0. *S*_*j*_(*t*) represents “adjacent neuron” set at the time *t*. With time increasing, the area of *S*_*j*_(*t*) continually reduces until the training procedure is completed.

**Step 2 (input vector).**

The vector *X* = (*x*_1_,*x*_2_,*x*_3_,…,*x*_*m*_)^*T*^ is fed into the input layer.

**Step 3 .**

Calculate the Euclidean distance among the input vectors and weight vectors of the output layer. The distance between the *j*th neuron in the output layer and the input vectors is described as follows:

(7) $$\begin{array}{c} d_{j}=||X-W_{j}||=\sqrt{\overset{m}{\underset{i=1}{\sum }}{\left(x_{i}\left(t\right)-w_{ij}\left(t\right)\right)}^{2}}, \end{array}$$

where *w*_*ij*_ is the weight between the neuron *i* in the input layer and neuron *j* in the mapping layer. The winning neuron can be obtained by calculation, which has a minimum distance j*. This is to say, with a certain unit *k*, *d*_*k*_ = min⁡(*d*_*j*_) exists for any *j*. Then its adjacent neurons sets are given.

**Step 4 (learning of the weights).**

The weights of the output neuron j* and the “adjacent neurons” are updated according to following equation:

(8) $$\begin{array}{c} \Delta w_{ij}=w_{ij}\left(t+1\right)-w_{ij}\left(t\right)=\eta \left(t\right)\left(x_{i}\left(t\right)-w_{ij}\left(t\right)\right), \end{array}$$

where *η* is a constant less than 1 and greater than 0, which gradually decreases to zero with the learning time.

**Step 5 .**

Calculate the output:

(9) $$\begin{array}{c} o_{k}=f\left(\underset{j}{\min}||X-W_{j}||\right), \end{array}$$

where *f*(∗) is usually a function from 0 to 1 or another nonlinear function.

**Step 6 (judge the termination conditions).**

If the requirement is achieved, end the algorithm. Otherwise, return to Step 2 and start the next round of learning.

## 4. SOM Neural Network Optimized by Improved PSO Algorithm

### 4.1. Basic Principle of PSO Algorithm

**Figure 8:**
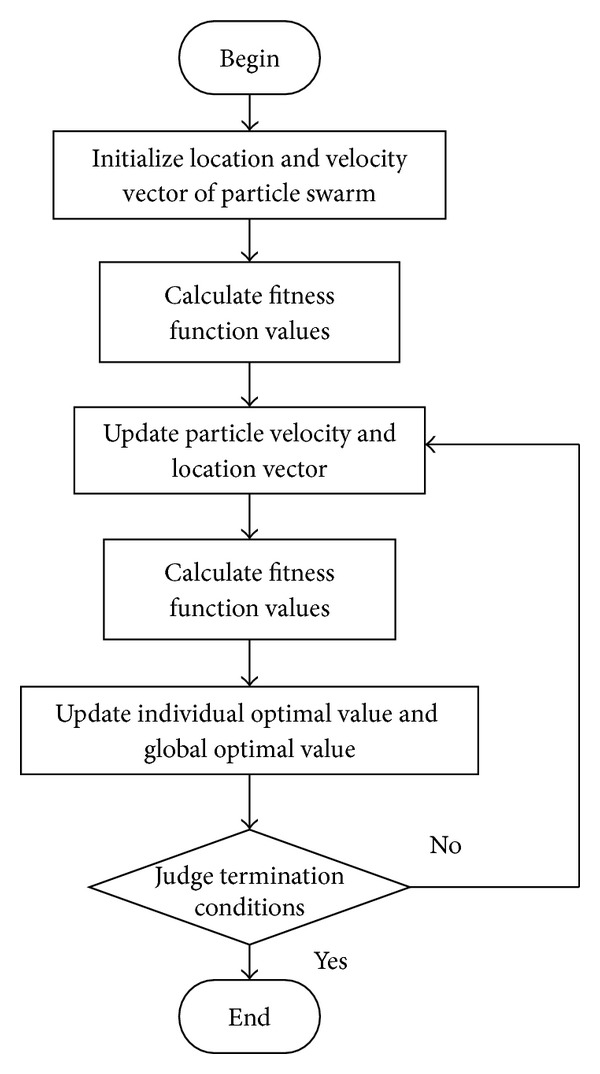
Flowchart of PSO algorithm.

Particle swarm optimization (PSO) algorithm is a kind of swarm intelligence heuristic algorithm, whose basic concept origins from the foraging behavior of birds flock [13–15]. In a *D*-dimension searching space, the population *X* = (*X*_1_, *X*_2_,…, *X*_*n*_) is composed of *n* particles. The *i*th particle is mapped to a *D*-dimension vector *X*_*i*_ = (*x*_*i*1_,*x*_*i*2_,*x*_*i*3_,…,*x*_*iD*_)^*T*^, which is the position of *i*th particle in the *D*-dimension search space, and also represents a potential solution of the discussed problem. According to the objective function, the fitness value corresponded by the position *X*_*i*_ of each particle can be calculated. The velocity of *i*th particle is represented as *V*_*i*_ = (*V*_*i*1_,*V*_*i*2_,*V*_*i*3_,…,*V*_*iD*_)^*T*^. The individual extreme is *P*_*i*_ = (*P*_*i*1_,*P*_*i*2_,…,*P*_*iD*_)^*T*^ and the global swarm extreme of population is *P*_*g*_ = (*P*_*g*1_,*P*_*g*2_,…,*P*_*gD*_)^*T*^. In the iteration process, the particles will update their velocities and positions according to

(10) $$\begin{array}{c} V_{id}^{k+1}=\omega V_{id}^{k}+c_{1}r_{1}\left(P_{id}^{k}-X_{id}^{k}\right)+c_{2}r_{2}\left(P_{gd}^{k}-X_{id}^{k}\right),\\ X_{id}^{k+1}=X_{id}^{k}+rV_{id}^{k+1}, \end{array}$$

where *ω* is the coefficient of keeping the original velocity, which is also called inertia weight. *c*_1_ is the weight coefficient of a particle tracking its history optimal value, which is called the learning factor, usually set as 2. *c*_2_ is the weight coefficient of a particle tracking the global optimal value, which is also called the learning factor, usually set as 2. *r*_1_ and *r*_2_ are random numbers uniformly distributed in region [0, 1]. *r* is a random number uniformly distributed in region [0, 1]. When updating the position of particles, a coefficient will be added in front of the velocity, which is called the constraint factor, whose acquiescent value is 1 [16, 17]. The basic procedure of the standard PSO algorithm is shown in Figure 8.

The training steps of PSO algorithm are described as follows.

**Step 1 (initialize particle swarm).**

Initialize the position vector and velocity vector of each particle, the inertial factor *ω*, the maximum permissible iteration steps, and the learning factors *c*_1_ and *c*_2_. Initialize the individual optimal value and global optimal value.

**Step 2 (calculate the fitness value of each particle).**

For each particle, the fitness value is compared with the individual optimal fitness value. If better, take it as the current best location.

**Step 3 .**

For all particles, the objective function value of the best position that they have experienced is compared with the global optimal fitness value. If better, take it as the current global optimal location. Update the particle's velocities and positions according to (10).

**Step 4 .**

Judge the termination conditions (the objective function reaches a certain value or the iteration number reaches maximum). If the termination condition is satisfied, the procedure is ended; otherwise, return to Step 2.

The fitness function is the basis that PSO algorithm guides the search direction. Therefore, it is very important to construct a suitable fitness function in the process of optimization. In this paper, the classification accuracy *ε*_*r*_ of SOM neural network is selected as the fitness function:

(11) $$\begin{array}{c} \varepsilon_{r}=\frac{m_{r}}{M_{r}}\times 100\%, \end{array}$$

where *M*_*r*_ is the number of the classification samples and *m*_*r*_ is the number of the right classification.

### 4.2. Improved Particle Swarm Algorithm

In order to improve the optimization speed and convergence precision of PSO algorithm, the domestic and foreign scholars put forward many adjustment methods of the inertia weight factor *ω*, such as the linear decreasing method and dynamic adjustment method [13, 18]. The advantage of the linear decreasing method is that the arithmetic is simple, easy to understand, and more convenient to implement. But its enlightening ability is weak. The dynamic adjustment method is just opposite. So the linear decreasing method is generally adopted under the condition that no high performance is required on the PSO algorithm. Generally, *ω* gradually decreases from 0.9 to 0.4.

**Figure 9:**
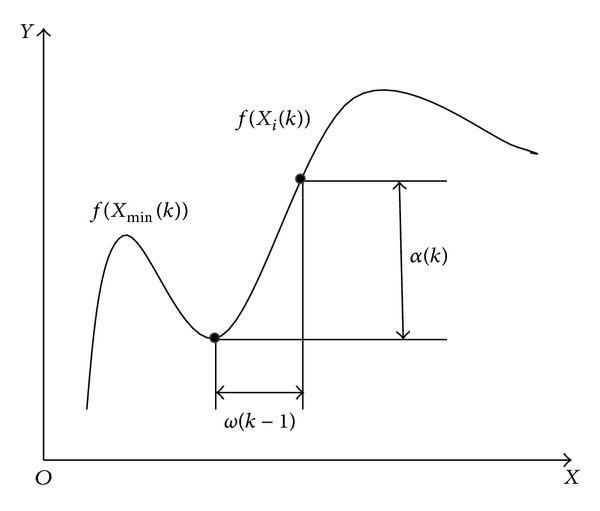
Dynamic adjustment method of inertia weight factor.

A new adaptive inertia weight approach is proposed which uses the success rate of the swarm as its feedback parameter to ascertain the particles' situation in the search space [19]. A new method of introducing nonlinear variation of inertia weight along with a particle's old velocity is proposed to improve the speed of convergence as well as fine-tune the search in the multidimensional space [20]. By analyzing the influence of two parameters describing the evolving state of the algorithm (the evolution speed factor and aggregation degree factor) on the PSO search ability, a new strategy is presented that the inertia weight dynamically changes based on the run and evolution state. In the strategy the inertia weight is given by a function of evolution speed factor and aggregation degree factor, and the value of inertia weight is dynamically adjusted according to the evolution speed and aggregation degree [21]. In this paper, a dynamic adjustment method relying on the flatness degree of the objective fitness function to change *ω* is adopted shown in Figure 9 [22].

Its advantage is to enhance the enlightening of the searching direction without increasing the calculation amount. *ω* is adjusted dynamically according to the following equations:

(12) $$\begin{array}{c} \omega \left(k\right)=1.1^{\lambda }\omega \left(k-1\right)\;\left(0<\omega \left(k\right)\leq 1\right), \end{array}$$

(13) $$\begin{array}{c} \lambda =\mathrm{sign}\left[\alpha \left(k\right)-\alpha \left(k-1\right)\right], \end{array}$$

(14) α(k)=1m∑i=1m|f(Xi(k))−f(Xmin⁡(k))|,(k=1,2,…,m),

(15) $$\begin{array}{c} f\left(X_{i}\left(k\right)\right)=f\left(x_{i,1}\left(k\right),x_{i,2}\left(k\right),\ldots ,x_{i,D}\left(k\right)\right), \end{array}$$

(16) $$\begin{array}{c} f\left(X_{\min}\left(k\right)\right)=\underset{i=1,2,\ldots ,m}{\min}f\left(X_{i}\left(k\right)\right), \end{array}$$

where *f*(*X*_*i*_(*k*)) is the calculated function of the *i*th particle at the iteration number *k*, *f*(*X*_min⁡_(*k*)) is the fitness function value of the optimal particle at the iteration number *k*, and the calculated *α*(*k*) is used to determine the flatness of objective function.

In the iteration process, if the calculated value of *α*(*k*) changes greater, it shows that the flatness of objective function changes little. Otherwise, it changes a lot. For each iteration, when *α*(*k*) changes, *ω*(*k*) also changes accordingly, which can make *ω* change following the changed searching locations. When [*α*(*k*) − *α*(*k* − 1)] < 0, it represents that the iteration is convergence, so the search step automatically becomes bigger in order to converge to the extreme point. When [*α*(*k*) − *α*(*k* − 1)] > 0, it shows that this iteration is diffuse, so the search step decreases automatically so as to realize the accurate search near the extreme value point.

### 4.3. Algorithm Procedure

**Figure 10:**
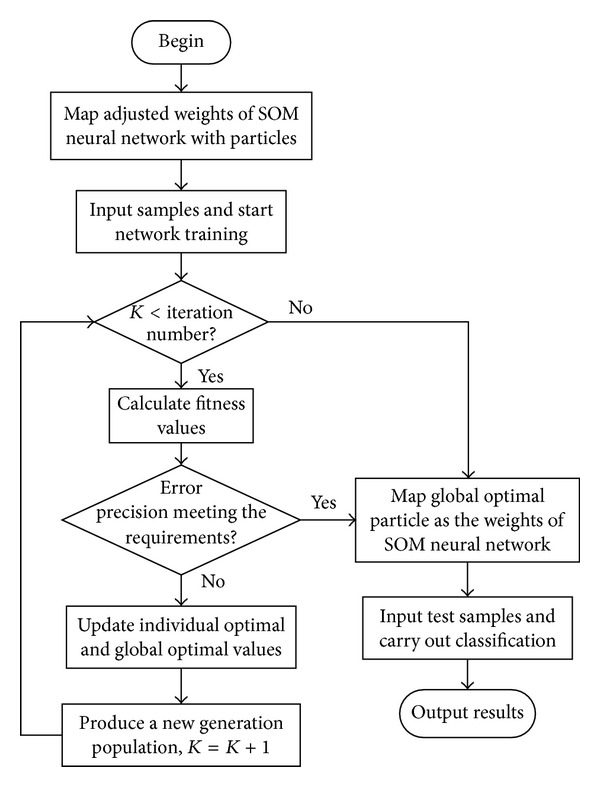
Flowchart of SOM neural network optimized by improved PSO algorithm.

In the learning process of SOM neural network, the weights of superior neighborhood directly affect the diagnostic accuracy of samples, so the PSO algorithm is used to optimize the weights of SOM neural network to make the weights of superior neighborhood achieve the optimal value. The flowchart of SOM neural network optimized by the PSO algorithm is shown in Figure 10. The steps of SOM neural network optimized by the PSO algorithm are described as follows.

**Step 1 (initialize particle swarm).**

Initialize the position and velocity vector of each particle, the inertial factor *ω*, the maximum permissible iteration number, and the learning factors *c*_1_ and *c*_2_. Initialize the individual optimal value and global optimal value.

**Step 2 (define the SOM superior neighborhood).**

Determine the optimized adjusted weights of all nodes in the superior neighborhood *N*_j*_(*t*):

(17) $$\begin{array}{c} w_{ij}\left(t+1\right)=w_{ij}\left(t\right)+\alpha \left(t,N\right)\left[x_{i}^{P}-w_{ij}\left(t\right)\right], \end{array}$$

where *α*(*t*, *N*) is a function of topological distance *N* between *j*th neuron of neighborhood and the winning neuron j* at the training time *t*  (*i* = 1,2,…, *n*; *j* ∈ *N*_j*_(*t*)).

**Step 3 .**

Adjusted weight is mapped to a particle in the particle swarm. Input the training samples and start training. When the training time is less than the set value, calculate the fitness. When it reaches the set value, the global optimal particle is mapped to the adjusted weights of SOM.

**Step 4 .**

After calculating the fitness, if error reaches the set value, the global optimal particle is mapped to the adjusted weights of SOM neural network. Otherwise, update the individual optimal value and the global optimal value.

**Step 5 .**

According to the PSO algorithm, produce a new generation population for the next training cycle.

**Step 6 .**

Finally, the optimal adjusted weights are obtained, with which the input testing samples are fed into the SOM neural network for fault classification test.

## 5. Simulation Research and Results Analysis

**Figure 11:**
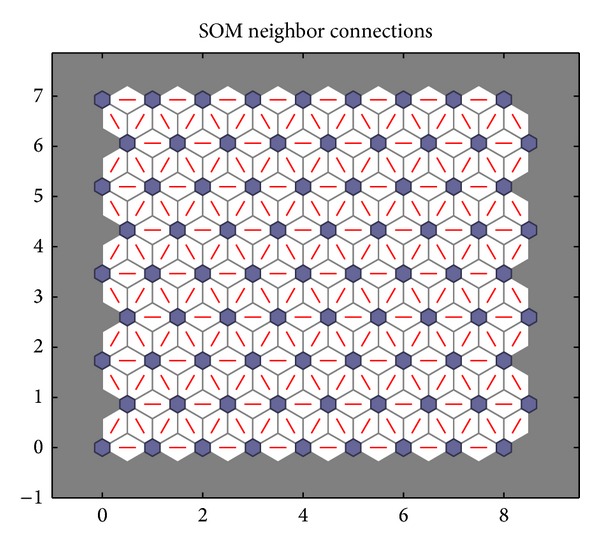
Topology structure of SOM neural network.

**Figure 12:**
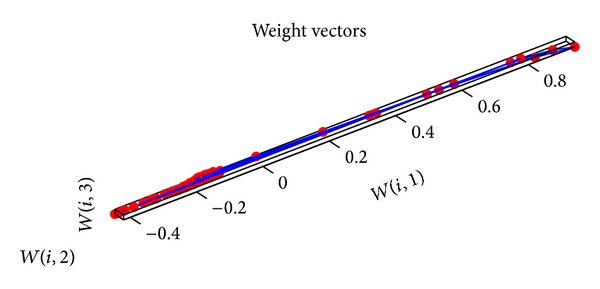
Training weights of SOM neural network.

**Figure 13:**
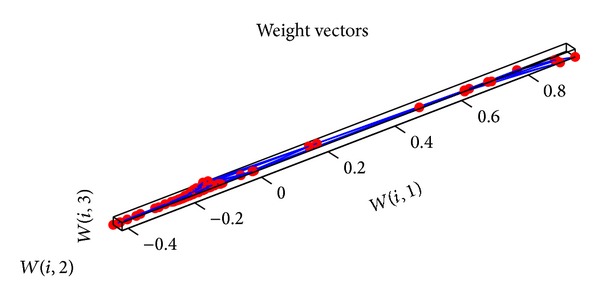
Training weights of SOM neural network optimized by PSO algorithm.

**Figure 14:**
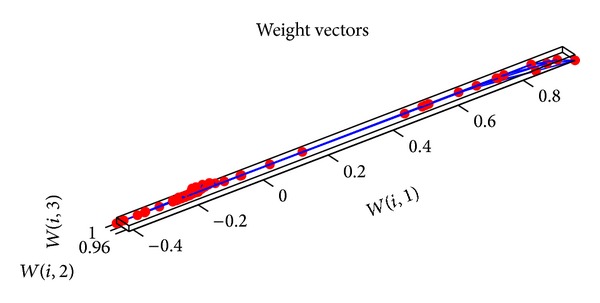
Training weights of SOM neural network optimized by improved PSO algorithm.

When the improved PSO algorithm is used to optimize the SOM neural network, the parameters should be initialized firstly. The initialization of PSO algorithm is described as follows. The number of particles is 100, the largest iteration number is 200, both *c*_1_ and *c*_2_ are 2, the minimum error rate is 0.0001, and *ω* is adjusted dynamically by (12)–(16). Then the input data are given a normalized disposal. The parameters of SOM neural network are set as follows. The input layer has 6 neurons. 9∗9 matrix is as the output for the competitive layer. The learning rate is 0.02, the neighborhood distance is 1, and the training number is 200. The topology structure of SOM neural network is shown in Figure 11. Every small blue hexagon represents a neuron in the competition layer and the red line represents the link of neurons. The initial neurons in the competition layer are equidistant. 300 groups of data are selected as the training samples and 80 groups of data are selected as the test samples. The training weights of standard SOM neural network are shown in Figure 12. The weights of SOM neural network optimized by the PSO algorithm are shown in Figure 13. The weights of SOM neural network optimized by the improved PSO algorithm are shown in Figure 14. Through contrasting these three kinds of training weights, it can be seen that the weights are more concentrated and the training performance becomes better.

The 300 training samples are classified as follows: 1~86 groups belong to the first fault type (motor fault), 87~159 groups belong to the second fault type (bearing fault), 160~238 groups belong to the third fault type (damage of sealing components), and 239~300 groups belong to the fourth type (running normally). The neurons in the competition layer are numbered from down to up, left to right. The neurons number increases gradually; that is to say, the neuron of lower-left corner is numbered as 1 and the neuron of top-right corner is numbered as 81.

**Figure 15:**
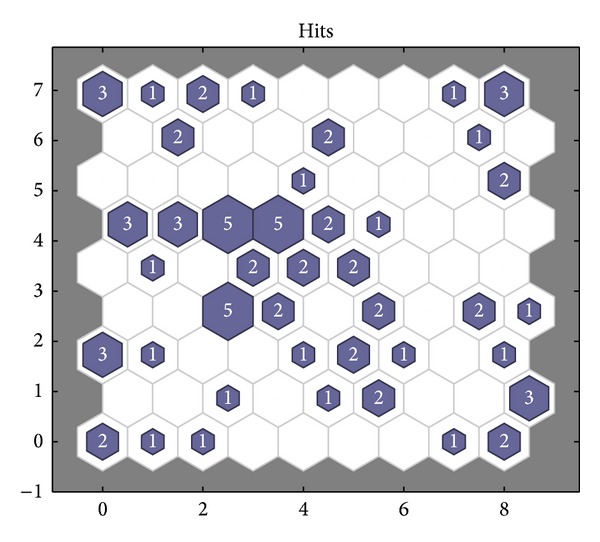
Statistics chart of winning neurons of SOM neural network.

300 groups of training samples, respectively, fall into the neurons in the competition layer by the simulation of SOM neural network. The winning neurons corresponding to every fault type are summarized based on MATLAB simulation results. The winning neurons corresponding to the first fault type are 55, 62, 63, 64, 69, 71, 72, 73, 74, 79, and 74. The winning neurons corresponding to the second fault type are 1, 2, 3, 10, 19, 22, 28, 30, 38, 39, 42, 46, and 50. The winning neurons corresponding to the third fault type are 7, 8, 9, 16, 17, 18, 26, 27, 30, 31, 40, 41, 42, 43, 48, 58, 59, and 67. The winning neurons corresponding to the fourth type are 6, 23, 24, 30, 31, 39, 40, 41, 47, 48, 56, 58, 65, 66, and 67. Neurons not having corresponding number are called “dead” neurons in that these neurons are at the suppressed state. There are 34 “death” neurons in Figure 15.

The number of winning neurons corresponding to the fault type is listed in Table 3 based on Figure 15. The comparison between actual values and predicted values of SOM neural network is listed in Table 4. It can be seen from Table 3 that the second, third, and fourth types of fault all having samples correspond to the winning neuron number 30, both the second and fourth types of fault having samples correspond to the winning neuron number 39, both the second and third types of fault having samples correspond to the winning neuron number 42, and both the third and fourth types of fault having samples correspond to the winning neurons numbers 31, 40, 41, 48, 58, and 67. If some test samples correspond to these winning neuron numbers, their fault type cannot be judged. It can be seen from Table 4 that in 80 groups of test samples only 66 numbers are determined and the fault type 0 cannot be diagnosed; that is to say, a total of 14 test samples cannot be diagnosed. Hence the fault diagnostic accuracy rate is 82.5%.

**Figure 16:**
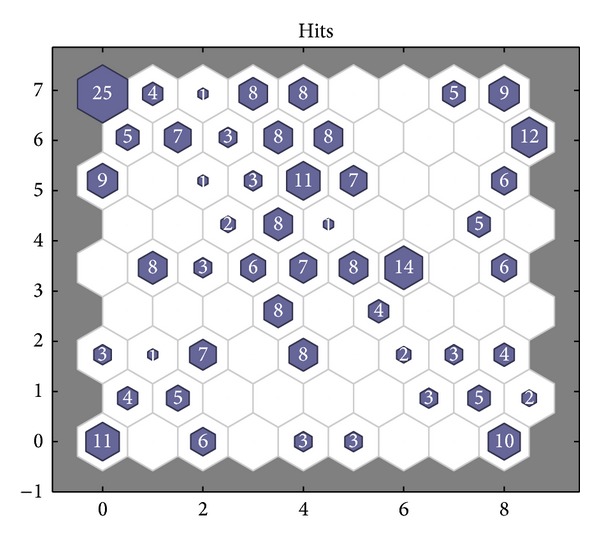
Statistics chart of winning neurons of SOM neural network optimized by PSO algorithm.

The statistics chart of winning neurons of SOM neural network optimized by PSO algorithm is shown in Figure 16. The number of winning neurons corresponding to fault class of SOM neural network optimized by PSO algorithm is listed in Table 5. The comparison between actual values and predicted values of SOM neural network optimized by PSO algorithm is listed in Table 6. It can be seen from Table 5 that the second, third, and fourth types of fault all having samples correspond to the winning neuron 5, both the second and fourth types of fault having samples correspond to the winning neurons 33, 48, and 49, both the third and fourth types of fault having samples correspond to the winning neuron 40, and both the second and the third types of fault having samples correspond to the winning neuron 50. But if test samples correspond to the winning neurons 5, 33, 40, 48, and 49 their fault types can not be judged. From Figure 16, there are 28 “death” neurons. Compared with Figure 15, the winning neurons have a great increase and the classification is more accurate. It can be seen from Table 6 that, in 80 groups of test samples, the fault type of 8 test samples cannot be determined, so the fault diagnostic accuracy rate is 90%.

**Figure 17:**
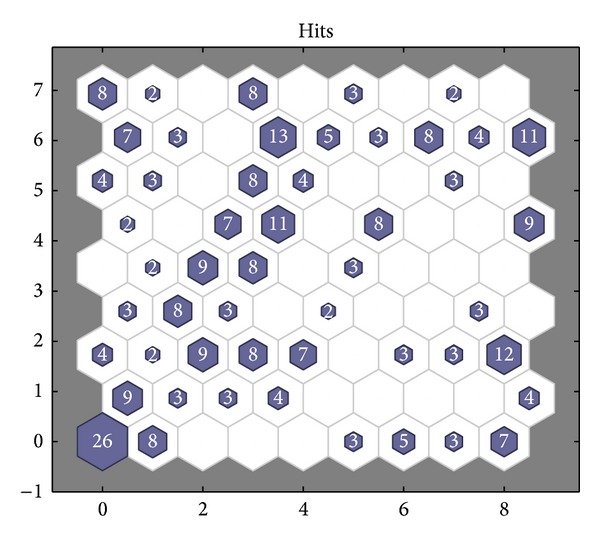
Statistics chart of winning neurons of SOM neural network optimized by improved PSO algorithm.

The statistics chart of winning neurons of SOM neural network optimized by the improved PSO algorithm is shown in Figure 17. The number of winning neurons corresponding to fault class of SOM neural network optimized by the improved PSO algorithm is listed in Table 7. The comparison between actual values and predicted values of SOM neural network optimized by the improved PSO algorithm is listed in Table 8. It can be seen from Table 7 that only the second and fourth types of fault having samples correspond to the winning neurons 41 and 50. Both the third and fourth types of fault having samples all correspond to the winning neurons 34 and 43. From Figure 17, there are 23 “death” neurons. Only four winning neurons have two types of fault and the training results are obviously much better compared with previous SOM and SOM optimized by PSO algorithm. It can be seen from Table 8 that, in 80 groups of test samples, four test samples cannot be determined with their fault types. In all test samples, no test samples correspond to the so-called “dead” neurons. Its diagnostic accuracy is 95%.

## 6. Conclusions

A fault diagnosis strategy of the polymerization kettle based on SOM neural network is put forward in this paper. Then the structure parameters of SOM neural network are optimized by the improved PSO algorithm. Finally, the simulation experiments of fault diagnosis are conducted combining with the industrial on-site historical data of the polymerization kettle and the simulation results show that the proposed PSO-SOM neural network fault diagnosis strategy is effective.

## Figures and Tables

**Table 1:** Main parameters of polymerization kettle.

Parameters	Unit	Parameters range	Fault type
Stirring speed	r/min	45 r/min~98 r/min	Motor fault
Stirring electric current	A	120 A~175 A	Motor fault
Polymerization kettle pressure	MPa	0.8 MPa~1.3 MPa	Shaft seal fault
Polymerization kettle temperature	°C	50°C~69°C	Shaft seal fault
Mechanical seal pressure	MPa	0.8 MPa~1.5 MPa	Damage of sealing components
Mechanical seal temperature	°C	55°C~80°C	Damage of sealing components

**Table 2:** Historical data of polymeric kettle.

Sample	Historical datum of polymerizer						Diagnosis type
	*a*	*b*	*c*	*d*	*e*	*f*	
1	56.71	128	1.90	54.96	0.98	58.84	2
2	56.67	160	0.82	55.16	0.98	60.75	4
3	113	128	0.86	54.51	0.97	58.75	1
4	56.67	136	0.80	54	2.6	58.75	3
5	56.75	192	0.82	55.13	0.99	59.36	1
6	56.77	137	0.82	79	1	59.52	2
⋮	⋮	⋮	⋮	⋮	⋮	⋮	⋮
380	56.48	146	0.83	55.49	1	109	3

**Table 3:** Number of winning neurons corresponding to fault class of SOM neural network.

Type	Number of winning neurons																	
I	55	62	63	64	69	71	72	73	74	79	81	0	0	0	0	0	0	0
II	1	2	3	10	19	22	28	30	38	39	42	46	50	0	0	0	0	0
III	7	8	9	16	17	18	26	27	30	31	40	41	42	43	48	58	59	67
IV	6	23	24	30	31	39	40	41	47	48	56	58	65	66	67	0	0	0

**Table 4:** Comparison between actual values and predicted values of SOM neural network.

Type	Number of winning neurons																	
1~18	79	24	81	48	50	41	16	42	6	79	17	22	24	19	73	1	39	65
Fault class	1	4	1	0	2	0	3	0	4	1	3	2	4	2	1	2	2	4
19~36	24	1	48	73	22	17	64	9	9	47	73	3	1	50	6	42	6	79
Fault class	4	2	0	1	2	3	1	3	3	4	1	2	2	2	4	0	4	1
37~54	58	22	42	72	73	40	22	30	74	39	24	10	23	8	1	18	47	55
Fault class	0	2	0	1	1	0	2	0	1	0	4	2	4	3	2	3	4	1
55~72	67	63	18	42	1	65	69	9	22	81	48	50	23	59	6	79	9	31
Fault class	0	1	3	0	2	4	1	3	2	1	0	2	4	3	4	1	3	0
73~80	27	26	1	24	19	27	1	73										
Fault class	3	3	2	4	2	3	2	1										

**Table 5:** Number of winning neurons corresponding to fault class of SOM neural network optimized by PSO algorithm.

Type	Number of winning neurons																		
I	1	2	10	11	20	37	46	55	56	64	73	74	75	0	0	0	0	0	0
II	5	8	9	15	16	17	18	24	26	27	33	36	41	42	48	49	50	58	67
III	5	13	23	32	40	41	50	54	60	62	63	70	77	79	80	81	0	0	0
IV	3	4	5	12	13	22	30	31	33	40	43	48	49	78	0	0	0	0	0

**Table 6:** Comparison between actual values and predicted values of SOM neural network optimized by PSO algorithm.

Type	Number of winning neurons																	
1~18	46	17	73	23	36	49	60	60	78	75	13	27	48	8	1	58	67	3
Fault class	1	2	1	3	2	0	3	3	4	1	3	2	0	2	1	2	2	4
19~36	3	27	23	1	50	63	11	81	81	22	1	58	67	48	78	60	78	75
Fault class	4	2	3	1	0	3	1	3	3	4	1	2	2	0	4	3	4	1
37~54	13	27	60	64	10	32	49	13	2	31	78	26	43	54	18	80	22	11
Fault class	3	2	3	1	1	3	0	4	1	4	4	2	4	3	2	3	4	1
55~72	5	55	41	67	67	3	37	41	33	73	23	67	12	60	78	75	81	40
Fault class	0	1	3	2	2	4	1	3	0	1	3	2	4	3	4	1	3	0
73~80	62	70	18	78	8	62	18	1										
Fault class	3	3	2	4	2	3	2	1										

**Table 7:** Number of winning neurons corresponding to fault class of SOM neural network optimized by improved PSO algorithm.

Type	Number of winning neurons																	
I	45	55	56	63	64	65	71	72	73	79	80	81	0	0	0	0	0	0
II	5	6	7	8	9	13	14	16	17	18	23	24	27	32	33	41	50	67
III	1	2	4	5	10	11	19	28	34	37	38	43	51	60	68	77	0	0
IV	12	23	30	31	34	40	41	42	43	48	49	50	57	58	59	66	75	76

**Table 8:** Comparison between actual values and predicted values of SOM neural network optimized by improved PSO algorithm.

Type	Number of winning neurons																	
1~18	79	41	72	60	23	23	5	5	12	45	68	33	23	16	73	14	13	75
Fault class	1		1	3	2	4	3	3	4	1	3	2	4	2	1	2	2	4
19~36	23	18	60	73	24	19	65	1	1	59	73	14	13	23	34	5	12	45
Fault class	4	2	3	1	2	3	1	3	3	4	1	2	2	2		3	4	1
37~54	68	33	5	81	64	51	24	42	55	30	30	8	31	19	9	10	59	65
Fault class	3	2	3	1	1	3	2	4	1	4	4	2	4	3	2	3	4	1
55~72	58	80	1	5	13	75	45	43	41	72	60	23	57	5	12	45	2	23
Fault class	4	1	3	2	2	4	1			1	3	2	4	3	4	1	3	4
73~80	28	37	9	30	16	28	9	64										
Fault class	3	3	2	4	2	3	2	1										
